# Supplementary material for: Subunit specialization in AAA+ proteins and substrate unfolding during transcription complex remodeling
Source: Proc Natl Acad Sci U S A. 2025 Apr 24;122(17):e2425868122. doi: 10.1073/pnas.2425868122 (PMC12054792; doi:10.1073/pnas.2425868122)
Supplement: Supplementary file 1 — Appendix 01 (PDF) [file pnas.2425868122.sapp.pdf]

Supplementary Information

Sample	Pixel Size (Å/pixel)	Total Dose ($e^-/Å^2$)	Defocus Range (µm)	Total Movies
RP(-11/-8)	1.1	50	-3 to -1	11 634
RP(-10/-1)	1.1	40	-3 to -1	15 534

Sample	RP(-11/-8) conformation 1	RP(-11/-8) conformation 2	RP(-11/-8) conformation 3	RP(-11/-8) conformation 4	RP(-11/-8) conformation 5	RP(-11/-8) conformation 6	RP(-10/-1)	pre-RPo state
Non-hydrogen atoms	26960	26819	26953	26928	26832	26903	26931	19635
Protein residues	5154	5125	5153	5148	5128	5143	5165	3581
Nucleic acid residues	68	68	68	68	68	68	64	87
Ligands	5 Mg^{2+}	5 Mg^{2+}	5 Mg^{2+}	5 Mg^{2+}	5 Mg^{2+}	5 Mg^{2+}	5 Mg^{2+}	1 Mg^{2+}
	5 ADP	5 ADP	5 ADP	5 ADP	5 ADP	5 ADP	5 ADP	
	5 AF3	5 AF3	5 AF3	5 AF3	5 AF3	5 AF3	5 AF3	
B factors (Å2)								
Protein	191.59	273.75	225.37	397.12	595.25	882.62	349.09	105.54
Nucleic acid	461.16	289.8	438.31	640.15	235.94	284.5	385.72	296.67
Ligands	121.29	563.98	171.92	585.45	307.84	706.08	344.07	326.69
R.m.s. deviations								
Bond lengths (Å)	0.007	0.002	0.003	0.01	0.002	0.011	0.002	0.004
Bond angles (°)	1.032	0.641	0.655	0.758	0.653	0.904	0.543	0.857
MolProbity score	2.52	1.52	1.68	2.11	2.09	2.42	1.83	2.09
Clashscore	23.55	3.53	4.7	11.71	10.91	19.19	6.79	8.4
Poor rotamers (%)	-	-	-	-	-	-	-	-
Ramachandran plot								
Favored (%)	85	93.84	93.31	90.73	90.57	0.2	92.78	86.1
Disallowed (%)	0.43	0.04	0	0.12	0.08	0.86	0.02	0.84

Table S1. Data collection, image processing, model refinement and statistics.

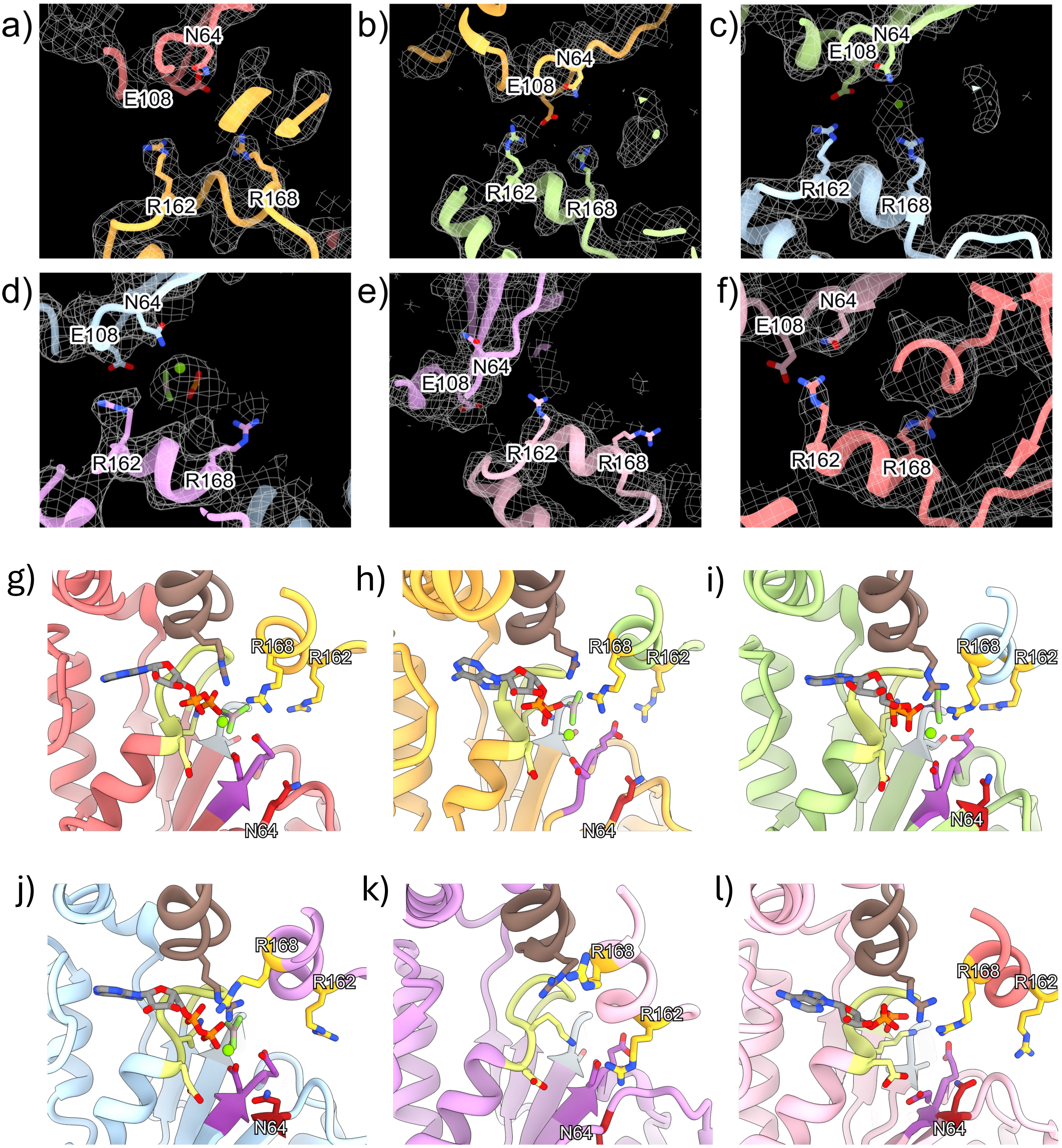

Fig. S1. nucleotide binding pockets of each protomer within the hexamer. (a)-(f) electron density for the key catalytic residues (g)-(l) positions of catalytic residues relative to nucleotides.

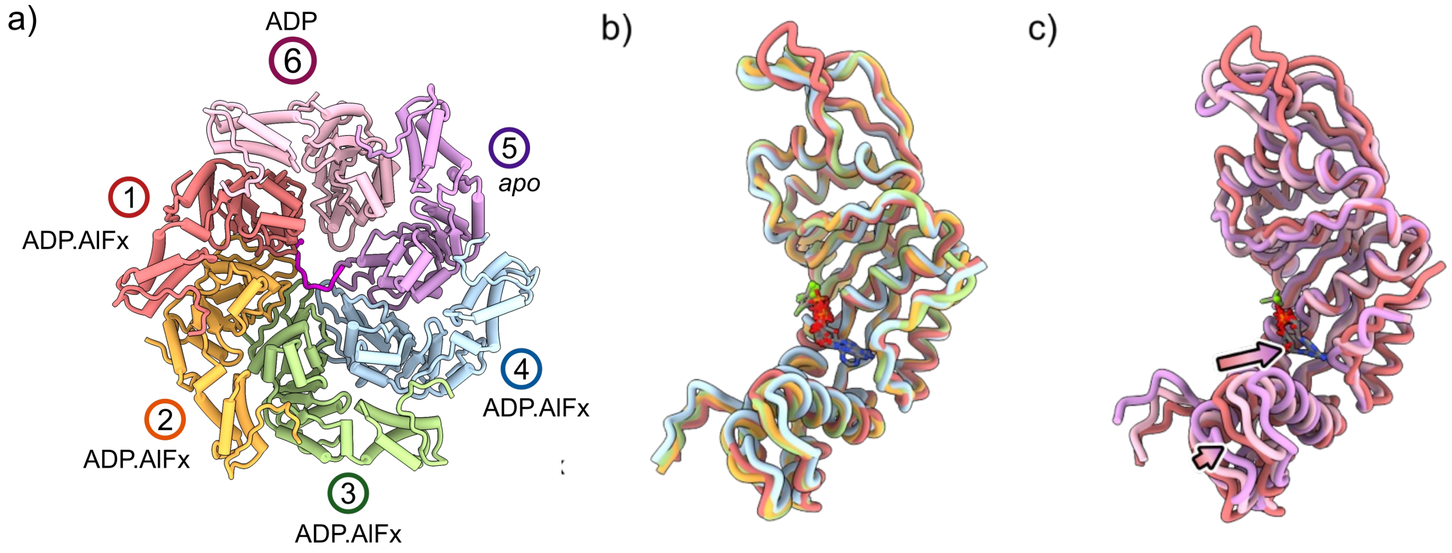

Fig. S2. Comparisons of PspF$_{1\text{-}275}$ protomers within the RPi(-12/-11) complex. (a) hexamer and their nucleotide states of each protomer. (b) Protomers 1-4 are similar in conformation. (c) comparisons of Protomer 1, protomer 6 and protomer 5 shows the conformational changes from ATP hydrolysis (ATP to ADP) and nucleotide release (ADP to nucleotide free).

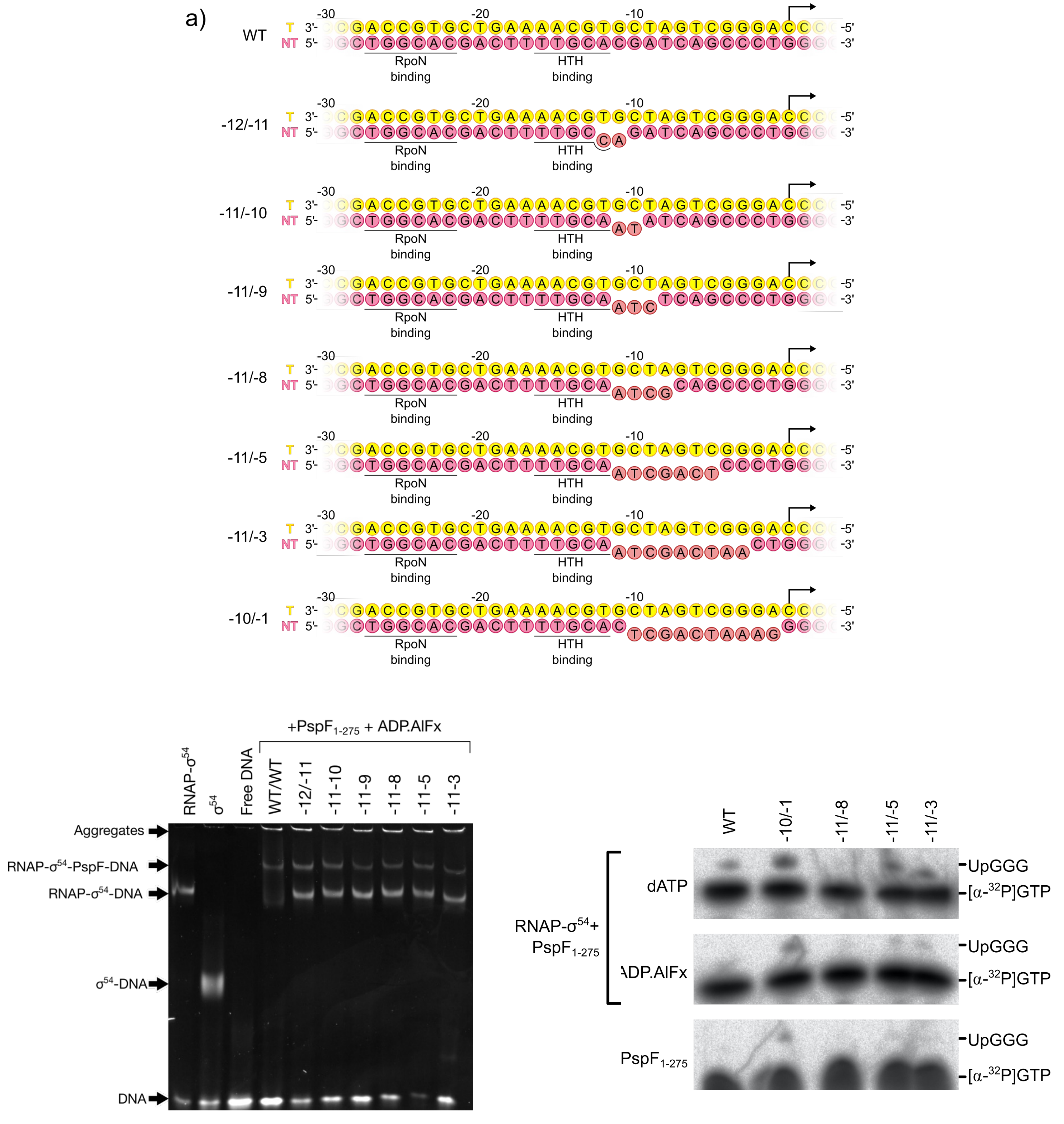

Fig. S3. Biochemical characterizations of intermediate complexes. (a) DNA susbtrates used to mimick progressively opening up DNA, (b) Native-PAGE gel of RNAP-σ^{54}-$PspF_{1\text{-}275}$ complexes trapped with ADP.AlFx and varying degrees of DNA mismatch. Lane labels indicate mismatched bases. For example, -11-9 indicates mismatched bases between -11 and -9. (c) ability of initiating transcription transcription in the presence of ADP.AlFx or dATP.

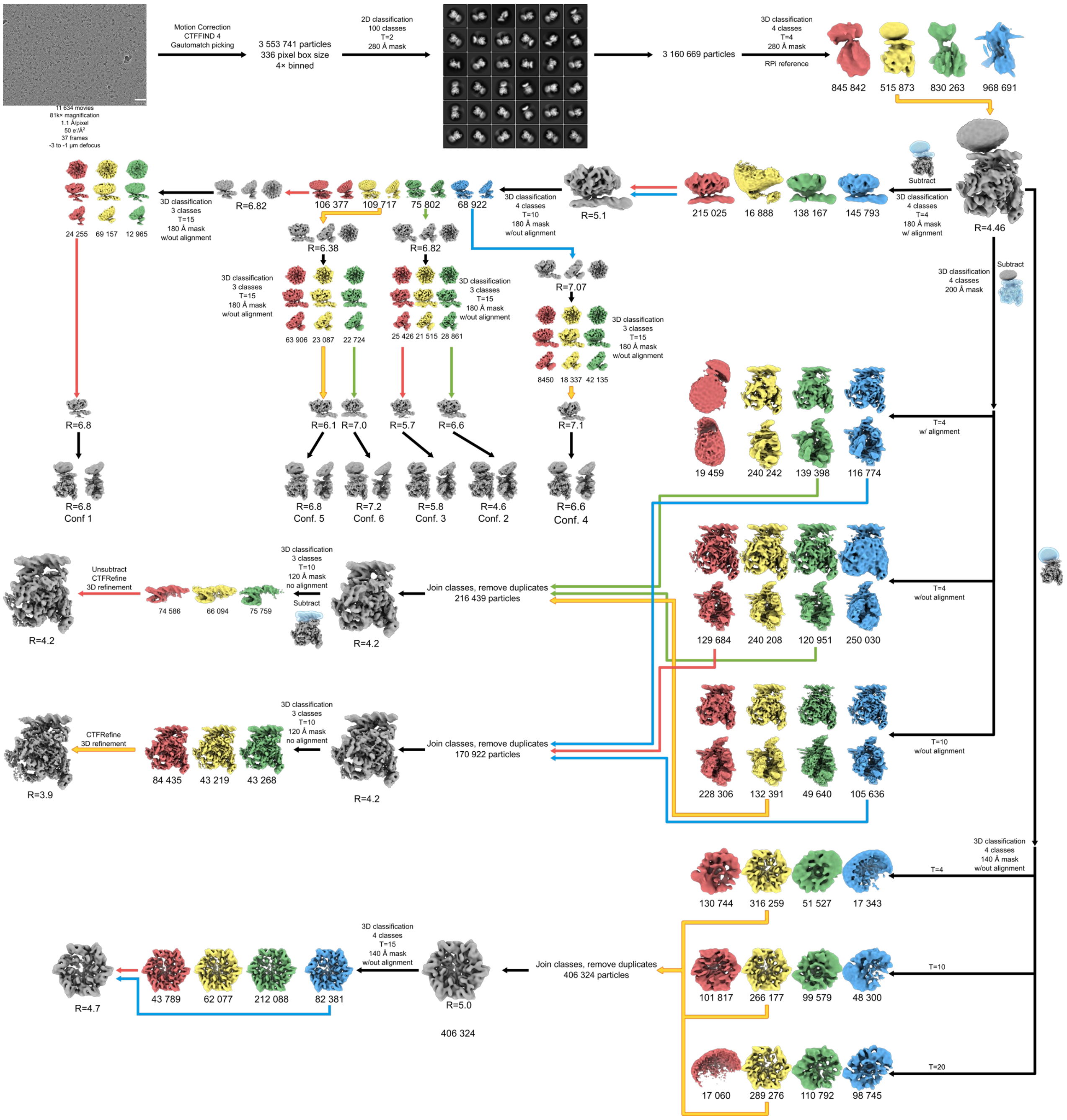

Fig. S4. image processing flow chat for RPi (-11/-8) complex.

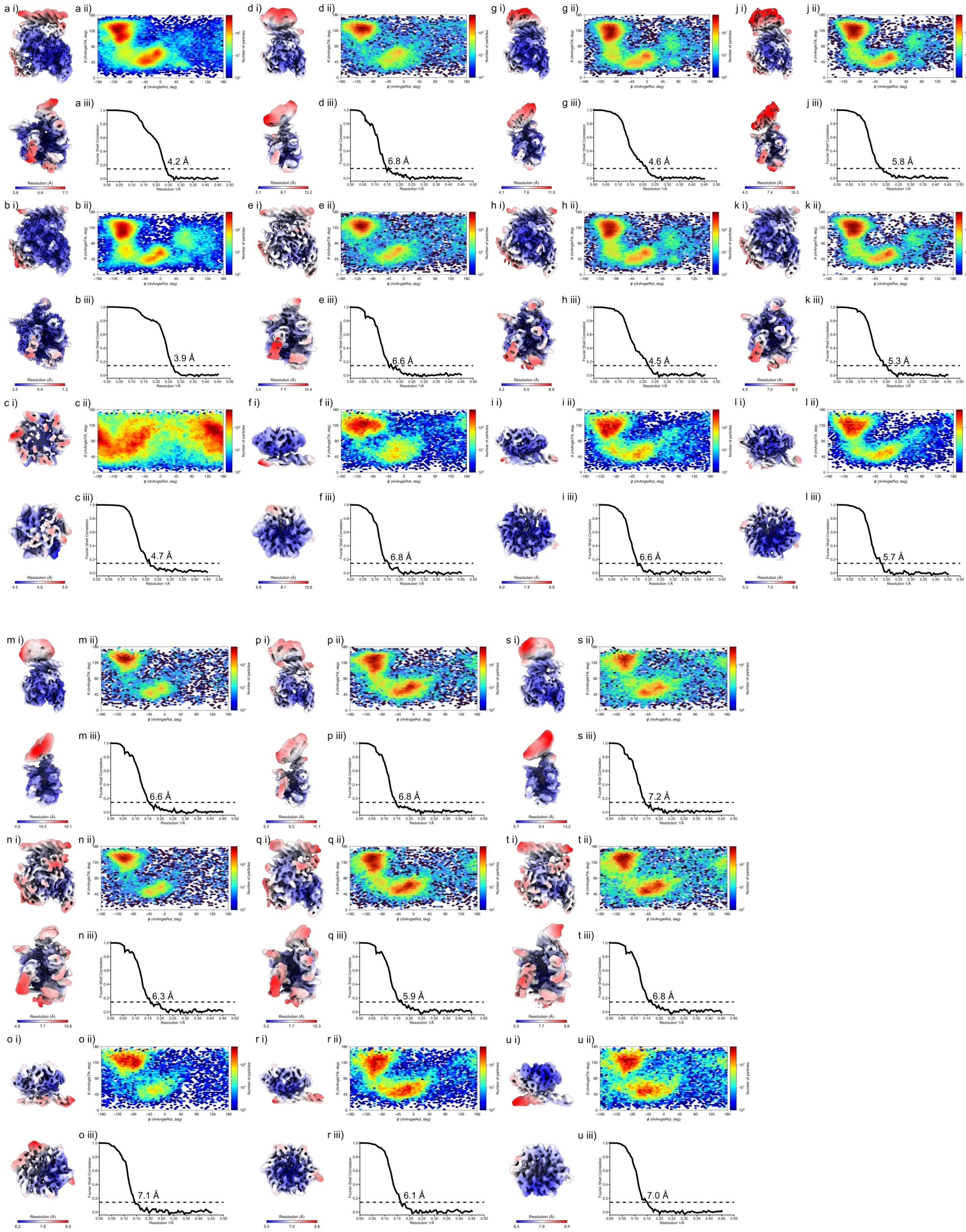

Fig. S5. **Local resolution map, angular distribution of particles and FSC curves of various reconstructions from RPi(-11/-8) complex.** a) open clamp and b) closed clamp c) and PspF maps obtained from subtracting the initial consensus map. Conformations 1 (d-f), 2 (g-i), 3 (j-l), 4 (m-o), 5 (p-r) and 6 (s-u) with the maps on the first row showing local resolution estimation of the global refinement, the second row obtained from subtracting the RNAP-σ^{54}-DNA density and running refinement and local resolution estimation, and the 3rd row obtained from subtracting the PspF-σ^{54}(excluding region II)-DNA density and running refinement and local resolution estimation. The subtracted refined maps were then used to create the composite maps shown in Supplementary figure 6. For each panel, i) shows the local resolution map ii) shows the angular distribution and iii) shows the FSC curves.

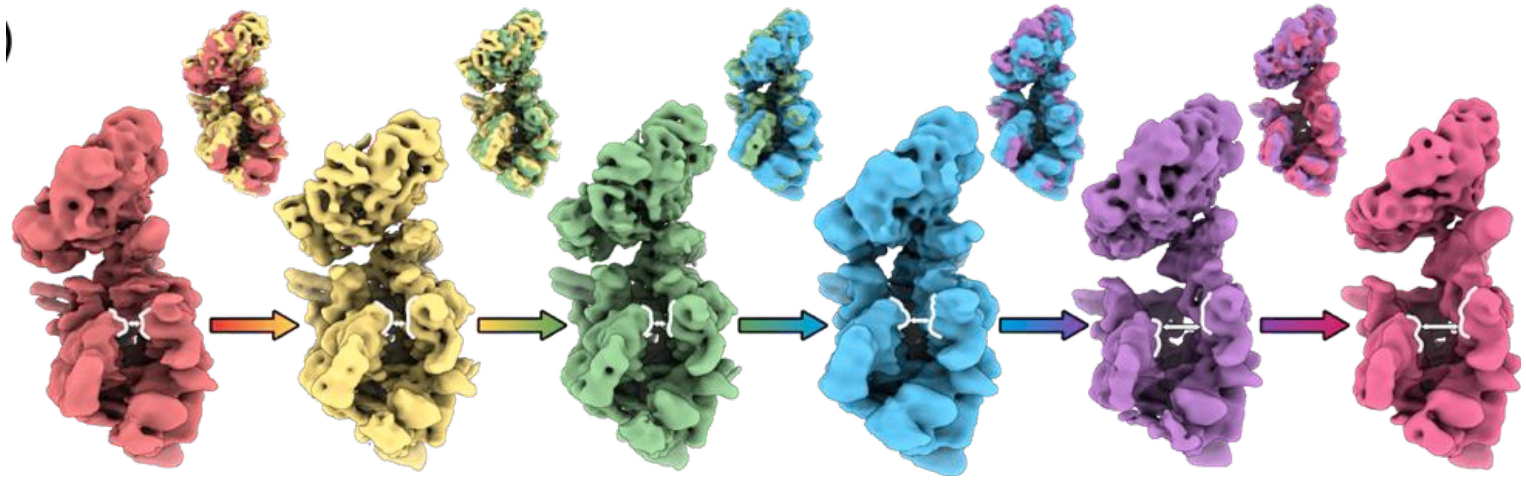

Fig. S6. Conformational heterogeneity of RPi (-11/-8), six well resolved conformations showing varying degrees of $PspF_{1\text{-}275}$ tilt and clamp opening.

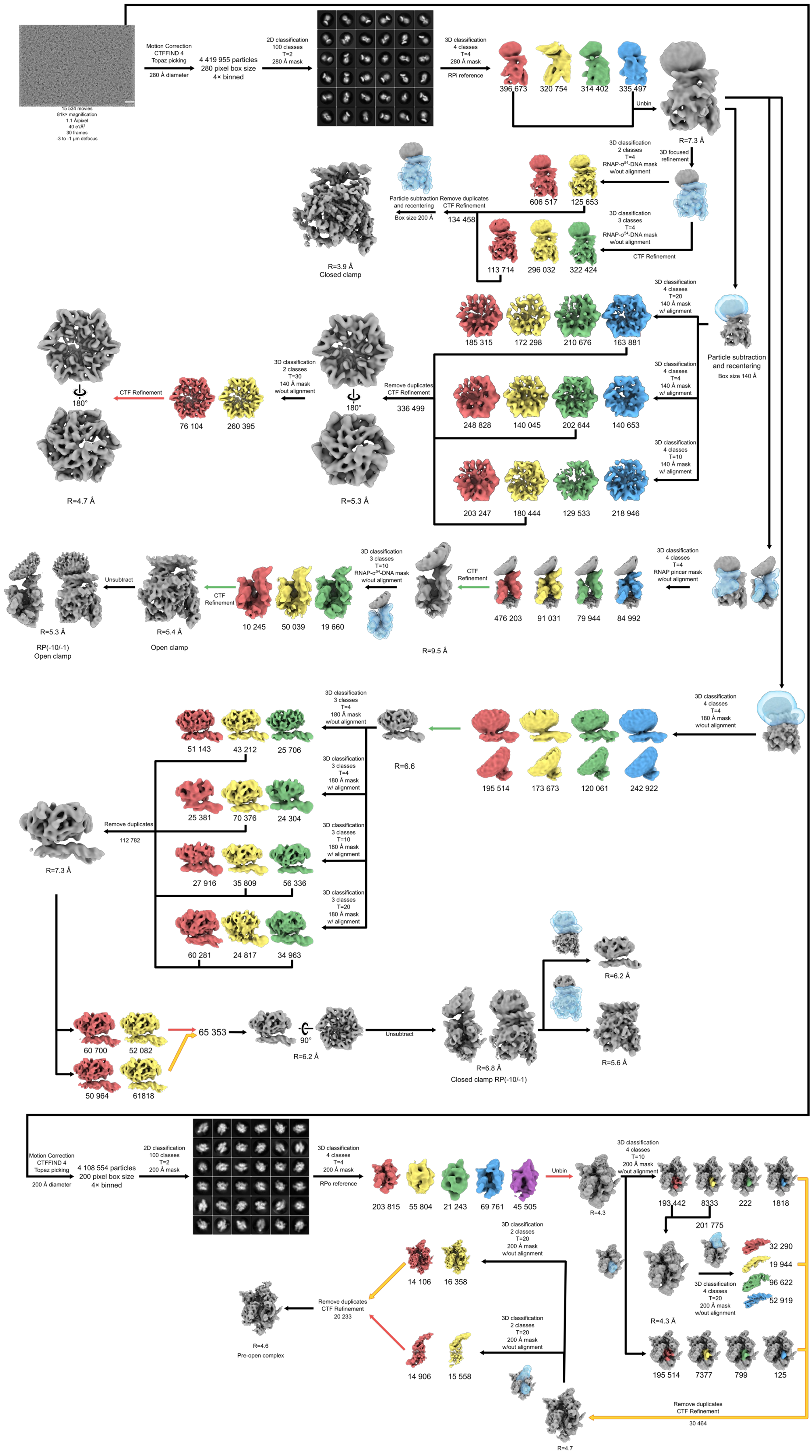

Fig. S7. Image processing flow chat of RPi (-10/-1) complex.

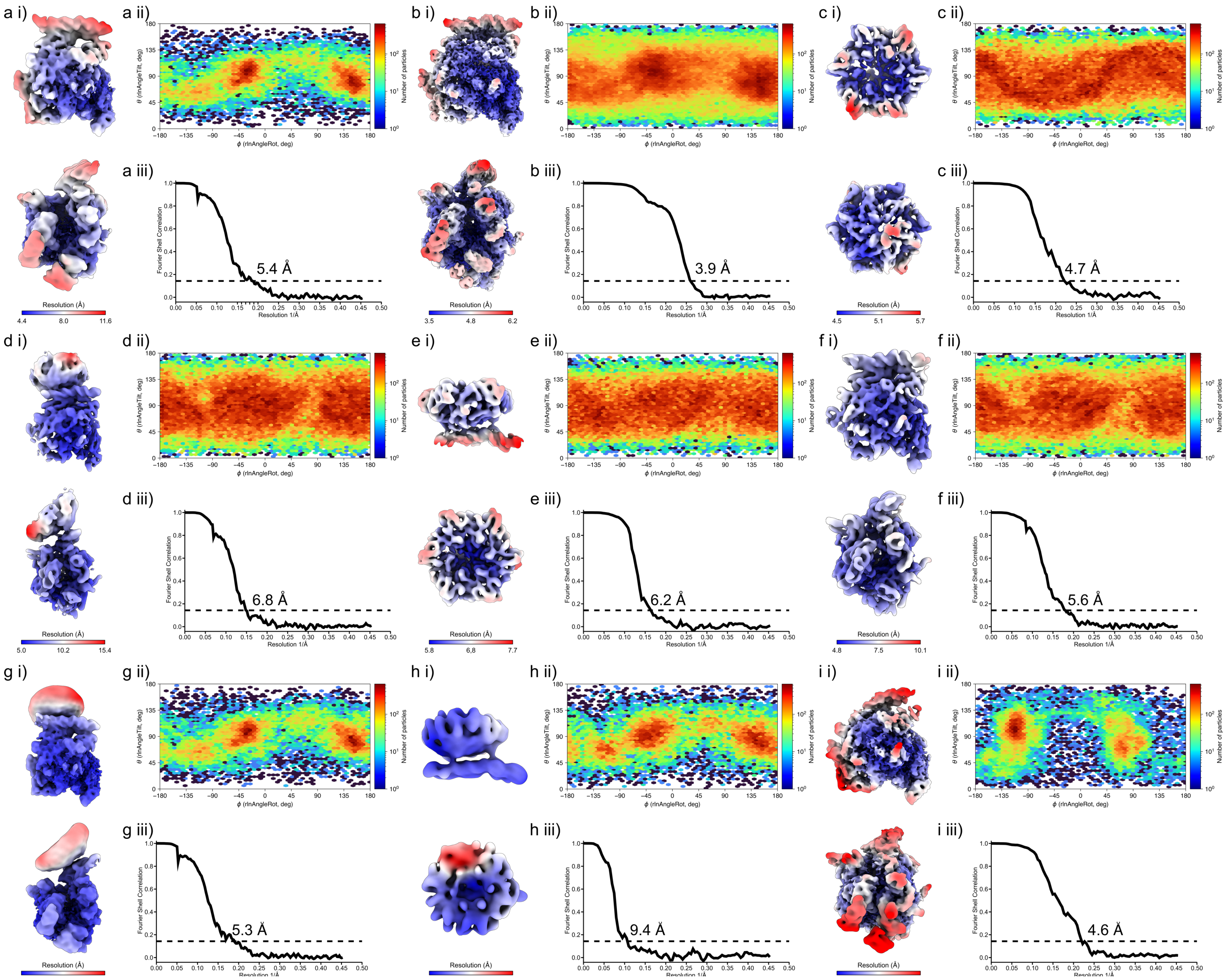

Fig. S8. Local resolution map, angular distribution of particles and FSC curves of various reconstructions from RPi(-10/-1) complex. a) open clamp and b) closed clamp c) and PspF maps obtained from subtracting the initial consensus map. d-f) closed clamp conformation obtained from reverting back to the original particle, whilst retaining the angular assignments the PspF- σ^{54}(excluding region II)-DNA density classifications and running global refinement and local resolution estimation (d), followed by focused classification, refinement and local resolution estimation of the PspF- σ^{54}(excluding region II)-DNA and RNAP-σ^{54}-DNA. g) Open clamp conformation obtained unsubtracting the RNAP β' clamp and β lobe - σ^{54}-DNA density classifications and running global refinement and local resolution estimation (g), followed by focused classification, refinement and local resolution estimation followed by focused classification, refinement and local resolution estimation of the PspF- σ^{54}(excluding region II) (h). i) Open complex densities. For each panel, i) shows the local resolution map ii) shows the angular distribution and iii) shows the FSC curves.

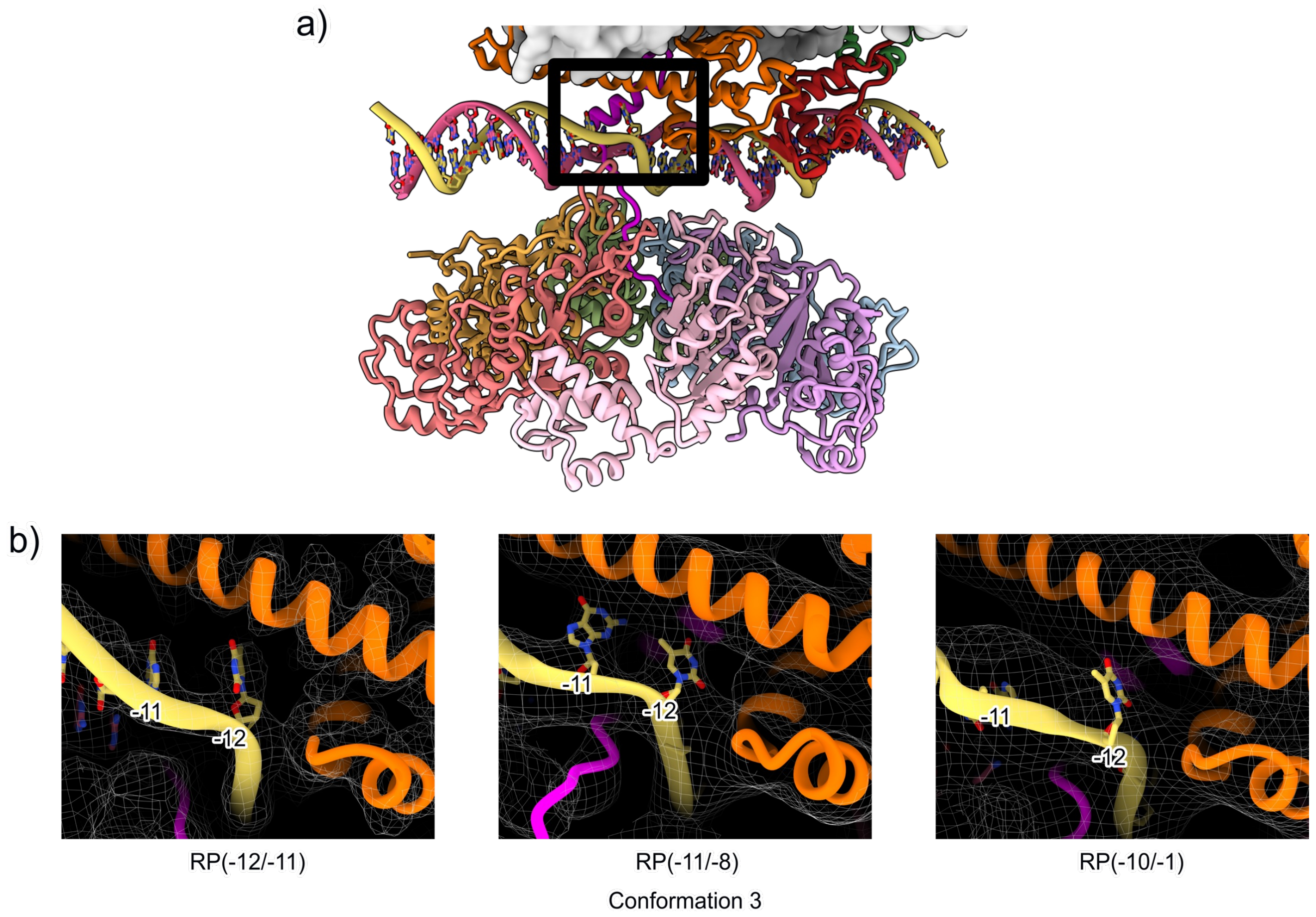

Fig. S9. Density of DNA at -12/-11 for RPi (-12/-11), RPi(-11/-8) and RPi(-10/-1).

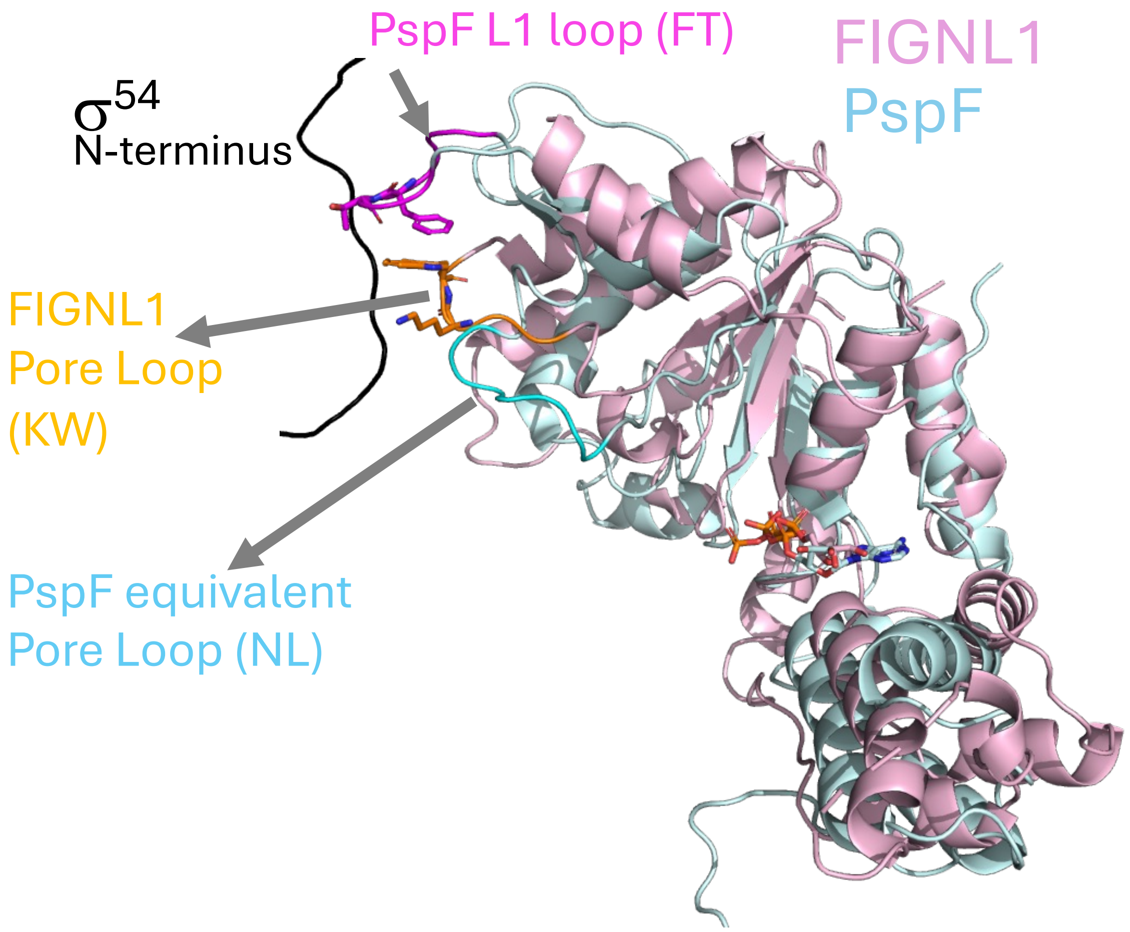

Fig. S10. Comparisons of the AAA+ domains of $PspF_{1\text{-}275}$ with that of FIGNL1. FIGNL1 pore loops are shown to interact and translocate substrates. PspF does not have residues which are conserved among AAA+ translocases. Instead L1 loops are positioned similarly to pore loops and perform similar roles.
